# Safety of Co-Administered Cannabidiol (CBD) and alcohol: a Phase I study

**DOI:** 10.1186/s42238-026-00457-1

**Published:** 2026-06-13

**Authors:** David Wolinsky, Sara Blaine, Justin C. Strickland, Erica N. Peters, Amy Harrison, Elise M. Weerts, Marcel O. Bonn-Miller

**Affiliations:** 1 Department of Psychiatry and Behavioral Sciences, Johns Hopkins University School of Medicine, Johns Hopkins Bayview Medical Campus, Behavioral Pharmacology Research Unit, 5510 Nathan Shock Drive, Baltimore, MD 21224 USA; 2 Department of Psychological Sciences, Auburn University, Auburn, AL USA; 3 Emerald Mountain Consulting, LLC, Charlottesville, VA USA; 4 University of Colorado Anschutz Medical Campus, Aurora, CO USA; 5 Charlotte’s Web, Denver, CO USA

**Keywords:** Cannabidiol, Alcohol use, Phase I, Hepatic safety

## Abstract

**Background:**

This Phase I randomized controlled trial aimed to assess the safety and tolerability of cannabidiol (CBD) and alcohol co-administration among moderate alcohol consumers in acute laboratory and chronic outpatient settings.

**Methods:**

Healthy adults ages 21–65 with moderate alcohol use completed three acute dosing sessions where they received either placebo, 50 mg CBD, or 100 mg CBD along with alcohol dosed to achieve peak blood alcohol concentrations (BAC) of 0.06 g/100 mL. Sessions were randomly ordered, and participants and outcome assessors were blinded to study drug during these laboratory sessions. Breath alcohol content (BrAC) and measures of subjective effects and impairment were collected from participants throughout each session. Participants then consumed 50 mg CBD twice daily for four weeks as outpatients while reporting alcohol use, craving, and adverse effects via a smartphone app. Metabolic labs and CBD content were measured from blood samples collected at four-week follow-up.

**Results:**

Twenty-nine participants were enrolled and nineteen participants completed the study. CBD was well-tolerated in both acute and chronic dosing sessions. Acute CBD-alcohol use was not associated with significant differences in subjective responses or breath alcohol content (BrAC) peak or time course compared to placebo for either CBD condition. Acute CBD-alcohol use was not associated with impairment 4 h after dosing. CBD use was not associated with significant changes in alcohol cravings or drinks per day, nor significant lab changes outside of decreases in protein and globulin, across the chronic dosing period.

**Conclusions:**

CBD co-administration with alcohol was not associated with significant changes in subjective or physiologic effects among moderate alcohol consumers in acute laboratory and naturalistic outpatient settings. The safety of CBD use in patients with more severe alcohol use should be examined in future studies.

**Trial registration:**

This study was registered at ClinicalTrials.gov on April 7th, 2022 under the code NCT05317507.

**Supplementary Information:**

The online version contains supplementary material available at 10.1186/s42238-026-00457-1.

## Background

Cannabis and alcohol represent two of the most commonly used psychoactive substances in the United States (2022 S. National Survey on Drug Use and Health (2022) detailed tables 2023), and are often used together (Yurasek et al. 2017; Gonçalves et al. 2023). Available studies have shown that co-use of cannabis and alcohol is associated with negative consequences such as performance impairment and a greater likelihood of developing a substance use disorder (Yurasek et al. 2017; Gunn et al. 2022). Most of these findings are isolated to the impact of the entire cannabis plant with a focus on delta-9-tetrahydrocannabinol (THC), its primary psychoactive constituent (Metrik et al. 2026; Hartman et al. 2015; Lukas and Orozco 2001; Ballard and Wit 2011). The effects of co-use of alcohol and cannabidiol (CBD)—another prominent cannabinoid that has not been associated with abuse liability and has demonstrated evidence of therapeutic effects for conditions including epilepsy syndromes and symptoms associated with movement disorders such as Parkinson’s Disease (Hsu et al. 2026; Solmi et al. 2023; Elsaid et al. 2019)—has received comparatively less attention.

A limited number of studies have directly examined CBD-alcohol interactions, and those studies have yielded mixed results regarding alcohol’s pharmacokinetics and subjective effects. One early study by Consroe et al. (1979) found that co-administration of oral CBD and alcohol (200 mg CBD and 1 g/kg alcohol) was associated with significant reductions in breath alcohol concentration (BrAC) compared to alcohol alone. Another finding from the Consroe study was that CBD and alcohol co-administration was associated with significant impairments in motor and psychomotor performance compared to placebo and CBD conditions but not alcohol alone, suggesting alcohol drove observed impairments. Additional studies by Belgrave et al. (1979) and Bird et al. (1980) involving co-administration of CBD dosed at 320 μg/kg with alcohol (0.54 g/kg) did not observe any effects of CBD on subjective intoxication or psychomotor performance when combined with alcohol. No differences between breath alcohol levels with CBD-alcohol co-administration were observed in either the Belgrave et al. or Bird et al. study, though it bears noting these studies involved smaller doses of CBD and alcohol than those used in the work by Consroe et al. Most recently, in a placebo-controlled cross-over human laboratory study, Karoly et al. (2023) evaluated BrAC and pharmacodynamics of CBD and alcohol co-administration when two different doses of CBD (30 mg and 200 mg) as well as placebo were administered just prior to a standardized drink of alcohol (0.6 g/kg vodka dissolved in orange juice). The rates of declines in BrAC differed in a dose-dependent manner such that placebo was associated with the steepest negative slope and 200 mg CBD was associated with the flattest slope. Additionally, 200 mg CBD was associated with slower reductions in stimulation while 30 mg CBD was associated with slower reductions in sedation as measured by the Biphasic Alcohol Effects Scale (BAES). Though these differences were statistically significant, the small magnitude of the differences and the limited precision of the results (as indicated by wide Bayesian intervals) led authors to conclude that any effects of CBD on BrAC and alcohol’s subjective effects are marginal. Additional research is needed to clarify these somewhat conflicting results on the acute effects of CBD-alcohol co-administration.

Although some previous studies have evaluated the effects of CBD-alcohol co-administration acutely in laboratory settings, the impact of CBD and alcohol co-use over longer periods of time, as would occur in real-world settings, has not yet been investigated. The longest duration over which alcohol use patterns in the setting of CBD were measured was five days as part of an observational study by Karoly et al. (2021) During this time, CBD-predominant cannabis was associated with significant decreases in number of drinking days compared to participants who used THC-dominant cannabis or cannabis with more equivalent amounts of THC and CBD. These results by Karoly et al. serve as a helpful contribution to the clinical literature covering the potential impact of CBD use on alcohol use behaviors (Gonzalez-Cuevas et al. 2018; Viudez-Martínez et al. 2018a, b; Moore et al. 2023; Turna et al. 2019), which has been proposed to arise from a number of mechanisms including mitigation of withdrawal symptoms (Dirik et al. 2026), reduced activation of the nucleus accumbens during alcohol exposure (Zimmermann et al. 2025), and attenuation of inflammation associated with alcohol craving (Karoly et al. 2020). However, this study’s results do not explore the tolerability and pharmacodynamic effects that would inform the safety of CBD-alcohol co-use in natural settings. Understanding these outcomes of CBD-alcohol co-use in a natural setting is a critical step that needs to be completed before CBD is considered for its impact on alcohol use patterns.

Metabolic effects—hepatic effects, in particular—represent another key measure of the safety of CBD and alcohol co-use that has not been evaluated in previous studies. Alcohol’s effects on the liver, including its capacity to cause short-term and long-term liver damage, are well-documented (Seitz et al. 2018) but the hepatic effects of CBD are far less clear. A number of preclinical studies identified evidence of liver damage following administration of CBD (Ewing et al. 2019; Henderson et al. 2023a), yet there is also evidence from preclinical work suggesting that CBD could serve as a therapy for certain forms of liver disease (Chen and Kim 2024). A recent systematic review of human studies found an association between CBD use and transient hepatic injury as indicated by elevations in the liver function tests (LFTs) alanine aminotransferase (ALT), aspartate aminotransferase (AST), and alkaline phosphatase. However, the majority of these cases involved doses of CBD > 300 mg or interactions between CBD and medications such as oral contraceptives and antiepileptics (Lo et al. 2023). It is currently unclear what effects, if any, CBD would have on the liver in the presence of co-administered alcohol in humans, and it is difficult to predict the nature of such effects. It is very possible CBD-alcohol co-use could lead to additive consequences for the liver; however, the preclinical literature finds that CBD administration can prevent mechanisms of alcohol-related liver injury such as hepatic steatosis and alcohol-mediated liver inflammation (Yang et al. 2014; Wang et al. 2017; Jiang et al. 2021). Enhancing the understanding of CBD’s impact on liver function and metabolic labs is of keen importance because of its expanding use in the general population, which includes people who regularly use alcohol.

The primary objective of the current Phase I, randomized, placebo-controlled study is to assess the safety and tolerability of co-administered CBD and alcohol among healthy adults with moderate drinking behaviors in both acute laboratory settings and a natural setting over a longer time period. The secondary objectives of this study include evaluating the subjective effects and alcohol-related effects arising from CBD and alcohol co-use. The CBD doses were determined as best reflecting the amounts that typical consumers use (Moltke and Hindocha 2021; Kaufmann et al. 2023) while remaining within the maximum amount that is recommended for safe use among healthy adults (Henderson et al. 2023b). By providing a comprehensive overview of the response to CBD and alcohol use in a healthy population with regular alcohol use, results from the study will yield important safety data that could guide consumer purchases and future regulatory decisions pertaining to CBD.

## Methods

### Study design

**Fig. 1:**
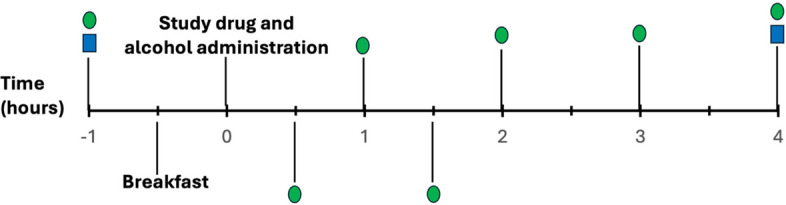
Acute dosing session schedule. Schematic of acute experimental session. Each marker indicates 0.5 h. Green circles indicate breath alcohol concentration measurement and administration of the Biphasic Alcohol Effects Scale (BAES), Alcohol Urge Questionnaire (AUQ), and Subjective Effects Questionnaire. Blue squares indicate administration of DRUID test and collection of blood sample to measure comprehensive metabolic panel

This randomized, double-blind, placebo-controlled study evaluated the safety, tolerability, and pharmacodynamic effects of oral CBD and alcohol co-administration. This study consisted of two phases: an acute experimental phase and a chronic outpatient phase. The experimental phase consisted of three visits to the Auburn University Research Facility with the co-administration of alcohol and CBD at two doses (50 mg and 100 mg) and placebo to allow for measurement of pharmacodynamic changes with acute co-administration. Please refer to Fig. 1 for the schedule of acute experimental sessions.

Following completion of the experimental visits, participants self-administered CBD over a four-week period in their individual home environment, where they reported levels of alcohol use, alcohol cravings, and adverse effects in a daily diary design. Participants provided blood samples for additional lab work at the end of the four-week period.

All procedures were conducted in accordance with the International Conference on Harmonization Good Clinical Practice Guidelines, the Declaration of Helsinki, and local laws and regulations. The protocol was approved by the Auburn University Institutional Review Board (IRB #20221017). The study was registered on https://clinicaltrials.gov (NCT05317507). No patient or public involvement occurred in the design, conduct, and reporting of the trial. The full study protocol and statistical analysis plan can be accessed at the website of the AUBIE lab led by Dr. Sara Blaine, PhD (https://cla.auburn.edu/aubie-lab/). The R&D arm of Canopy Growth Corporation was closed resulting in discontinuation of study recruitment and lower accrual of particpants than originally planned*.* No other changes to the trial, including measured outcomes and analyses, occurred after the trial commenced. Reporting of trial methods adhered to CONSORT reporting guidelines, and the completed CONSORT checklist is provided as Additional File 1.

### Participants

Medically healthy adults ages 21–65 who drank alcohol were recruited from social media and local advertisements. Participants were also recruited from past studies with similar inclusion criteria (described below). Interested individuals completed a Qualtrics survey to confirm initial eligibility. Individuals deemed eligible based on the initial survey completed an in-person screening exam where they provided blood and urine samples for laboratory chemistries including urine drug screens, urine pregnancy tests, and comprehensive metabolic panels (CMPs), and completed assessments for alcohol use including the Timeline Followback (TLFB) and an adapted version of the Alcohol Use Disorders Identification Test (AUDIT). The Daily Drinking Questionnaire (DDQ) (Collins et al. 1985) was used to collect information on individual alcohol use patterns and demographics, including height and weight, to calculate average blood alcohol concentrations (BAC) prior to enrollment. Specifically, BAC was calculated using the Widmark formula (available at https://www.calculator.net/bac-calculator.html) based on body weight, sex, alcohol consumed (amount and ABV%), and estimated drinking time. DDQ results needed to indicate drinking sufficient amounts of alcohol to reach an average BAC of at least 0.06% over the past month for an individual to be included in the study. Individuals were excluded if they met any of the following study criteria: (1) were pregnant or breastfeeding, (2), were a woman of childbearing potential unwilling to use contraception from 21 days prior to study medication administration to 28 days afterwards, (3) historically or currently meeting DSM-5 criteria for severe Alcohol Use Disorder (AUD) as determined using the Structured Clinical Interview for DSM-5 (SCID-5), (4) had a history of epilepsy, hepatitis, clinically significant hepatic or renal impairment, human immunodeficiency virus (HIV), or a significant psychiatric disorder, (5) used hepatotoxic medications or medications that interact significantly with CBD, (6) changed their use of over-the-counter or prescription medication within 28 days of screening or during the study, (7) had any allergy or sensitivity to any compound related to cannabis, (8) had consumed grapefruit products in the week prior to intake, (9) used any cannabis or hemp products and analogues in the 28 days prior to screening, (10) participated in any other clinical trials within 30 days prior to screening visit, (11) endorsed suicidal intent at the screening visit, (12) had a positive breath alcohol test or urine drug test at screening visit or any experimental visits, (13) self-reported use of nicotine or nicotine replacement products at screening visit, (14) had a past or current diagnosis of a psychiatric disorder or substance use disorder, (15) positive results on alcohol breath test or urine drug screen for benzodiazepines, PCP, barbiturates, antidepressants, cocaine, amphetamine, methamphetamine, THC, or opioids at screening visit or at any experimental visit, (16) demonstrated behavior indicating they would be unable to comply with the requirements of the protocol, or (17) had clinically significant conditions or abnormal findings that would preclude study participation.

### Acute experimental sessions

Individuals deemed eligible to participate in the study were scheduled to begin their three experimental visits within two weeks of screening. On arrival to each visit (scheduled to begin at 11:00 AM), participants were served a standard breakfast meal (i.e., Jimmy Dean™ Breakfast Bowl), provided breath samples to test BrAC using the Draeger Alcotest 6820 breathalyzer, provided urine for drug screens and pregnancy tests (if biologically female) and provided bloodwork for pre-dose measures of metabolic labs and CBD and metabolites. Approximately thirty minutes later, participants consumed two softgels. Each softgel contained either 50 mg CBD isolate in medium-chain triglyceride (MCT) oil (active) or MCT oil with no CBD (placebo) and were identical in appearance. Softgels were manufactured for the sponsor according to the Current Good Manufacturing Practice (cGMP) Title 21 of the Code of Federal Regulations (CFR) Part 111 (21 CFR Part 111) by a contract manufacturer. At each session, participants received two softgels in a randomized, within-subject order: two placebo softgels (0 mg CBD), one placebo softgel and one CBD softgel (50 mg CBD), or two CBD softgels (100 mg CBD). Participants then immediately consumed alcohol (80 Proof Vodka with Crystal Light) that was dosed by body weight and biological sex to achieve a target peak BAC of 0.06 g/100 mL. This alcohol dose was selected because it has been found to best reflect moderate alcohol use, and the target alcohol level has been used in previous projects by the investigative team (Blaine et al. 2019; Spagnolo et al. 2014; Schultz et al. 2020). After consuming the alcohol beverage, participants completed self-report assessments and provided breath samples for BrACs every 30 min for 2 h, then every 60 min for another 2 h (i.e., 30 min, 60 min, 90 min, 120 min, 180 min, 240 min). Participants also had a blood sample collected at the 240-min time point for monitoring of metabolic labs and plasma concentrations of CBD and its metabolites. Between assessment times, participants could relax and were provided with access to laptops, IPads, TV, and streaming services and access to their phones.

All doses were administered in a double-blind, randomized fashion, with both participants and investigators blinded as to the dose of study product administered at each visit. To maintain the blind, study drugs were prepared in a blinded manner. Containers were labeled in a blinded and coded fashion ready for dispensing. Designated staff members were unblinded, but these members lacked access to site staff, study data base, or any other sources of information that could compromise study blind.

A statistician at Canopy Growth who was otherwise not involved in the project generated the random allocation sequence using a computer-generated random number sequence. Simple randomization that did not involve blocking, stratification, or adaptive methods was applied. Sealed envelopes containing the unblinded intervention sequence for each unique assigned study number were provided by the Sponsor and were only to be opened in the event of an emergency. Unblinded envelopes were stored in a saferoom that required ID card access.These envelopes were not opened until after the trial ended. A blinded version with the interventions labelled A, B, or C was used by study staff to direct dosing.

As oral CBD (100 mg) is undetectable in plasma 24 h after acute use (Bergeria et al. 2022), each visit was scheduled at least two days apart to avoid influence of residual effects of CBD.

### Acute experimental session measures

The Biphasic Alcohol Effects Scale (BAES) (Martin et al. 1993) is a self-report, unipolar adjective rating scale that is designed to measure both stimulant and sedative effects of alcohol. It consists of 14 items in which participants rate the extent to which drinking alcohol has produced these feelings at the present time on an 11-point scale from 0 (not at all) to 10 (extremely). Items are summed to yield two subscales: Stimulant and Sedative.

The Alcohol Urge Questionnaire (AUQ) (Bohn et al. 1995) consists of 8 statements about the respondent’s feelings and thoughts about drinking as they are completing the questionnaire (i.e., right now). Drinking refers to various types of alcohol, including beer, wine and liquor. The respondent is asked to respond to each statement about alcohol craving via a 7-item Likert scale ranging from “strongly disagree” to “strongly agree.”

The Subjective Effects Questionnaire consists of 14 items in which participants report their subjective states after consuming the study product and beverage. Participants are instructed to rate how they are feeling “right now” on 4 items specifically related to the study product and beverage, and to rate how much they are experiencing 10 adjectives. Items are rated on a 100-point visual analog scale, with anchors of “not at all” and “extremely.” See Additional File 2 for the complete Subjective Effects Questionnaire.

The DRiving Under the Influence of Drugs® (DRUID®) is a validated measure of cognitive and motor impairment that has been utilized in studies assessing impairment related to cannabis use (Karoly et al. 2022; Spindle et al. 2021) as well as co-use of cannabis and alcohol (Zamarripa et al. 2022). The DRUID measures reaction time, decision-making, hand–eye coordination, and time estimation under conditions of divided attention via an array of computerized tasks. Task 1 involves shapes (either square or circle) flashing across a screen, with one designated as the target shape and the other as the control shape. Participants are asked to touch the shape when the target shape appears and then the top of the screen when the control shape appears. This task assesses reaction time to touching the screen and number of errors. Task 2 involves participants estimating when 30 seconds have passed by pressing a button on the screen, and then touching the screen when shapes appear during this interval. Reaction time to shapes and accuracy of time estimation were assessed. Task 3 involves participants keeping their fingers on a circle while counting the number of squares that appear on a screen. Task 4 is a balance task in which participants stand on one leg while holding the IPad collecting DRUID data in the same hand, then switch to the other leg and switch the device to their other hand. Performance data from each of the four tasks were integrated to yield a global impairment score—the primary outcome for the DRUID task.

The Biphasic Alcohol Effects Scale, Alcohol Urge Questionnaire, and Subjective Effects Questionnaire were all administered pre-dose and 30 min, 60 min, 90 min, 120 min, 180 min, 240 min following consumption of study drug and the alcohol beverage. The DRUID was completed four times during screening for practice, then once pre-dose and once at 240 min post-dose.

Adverse events were monitored throughout each experimental visit, recorded by study monitors, and reported to the investigators. Participants were discharged from the visit after BrAC was 0.02% or less, and rideshares were provided for all participants.

### Chronic dosing session

At the end of their third dosing session, participants were provided a four-week supply of 50 mg CBD softgels to be taken twice daily for a total of 100 mg of CBD a day. Participants were trained on what constitutes a standard unit of alcohol (1.5 oz of liquor, 5 oz of wine, 12 oz of beer). Data were recorded on the smartphone-compatible experience sampling application Metricwire, which has been used to measure cannabis use in previously published studies (Freisthler et al. 2026; Luken et al. 2024; Shrier et al. 2024). The following measures were collected by the Metricwire app: daily CBD use, daily alcohol use (reported as standard drinks per day), subjective alcohol intoxication (represented by a Visual Analog Scale from 0 to 10 with 0 indicating no intoxication and 10 indicating the most intoxication), adverse events (represented by self-report in which participants either answered “yes/no” to adverse events and then describing the event if they answered in the affirmative), and alcohol craving. Alcohol craving was measured via completion of the Penn Alcohol Craving Scale (PACS) (Flannery et al. 1999), a 5-item self-administered measure. Data were collected once every evening of the four-week study, with participants reporting on their behavior that day.

Within 24 h of finishing their at-home CBD supply, participants were asked to return to the laboratory to have their height, weight, BMI, and vitals collected. They were also asked to complete urine drug screens, urine pregnancy tests, blood draws to measure comprehensive metabolic labs and plasma concentrations of CBD and its metabolites and breathalyzer screens. Participants were also asked to give a final report on their assessment of AEs/SAEs and compliance with the study drug.

### Sample size justification

Precision analysis was performed to determine the sample size needed to achieve a suitable confidence interval width for estimating prevalence of ALT elevations among individuals using 100 mg CBD for four weeks. Based on previous research reporting ALT elevation prevalence rates between 0.03–11% among participants using CBD doses similar to or higher than those in the current study (Devinsky et al. 2017, 2018; Miller et al. 2020), a Monte Carlo simulation using 5000 simulated samples and an ALT prevalence of 10% to determine the sample size necessary to achieve at least 80% power to obtain full confidence interval widths < 0.25 was conducted. Based on this simulation, a sample size of 30 was found to yield 83% power to achieve the desired confidence interval width.

### Data analysis

Only participants who completed all three experimental visits and the entire chronic outpatient phase were included in the analysis. Peak effects from acute experimental sessions were evaluated using linear mixed effect models including the main effects of CBD dose and a random intercept for participant to account for the repeated measure design. Time course data were evaluated by also including a main effect and interaction of time in models evaluating change from baseline. Time to peak effect (Tmax) was calculated as the timepoint at which peak effect was observed. Outpatient data were evaluated using linear mixed effect models including a main effect of day and random participant intercept to evaluate changes over the 4-week period. Self-reported adverse events from the outpatient period were descriptively tabulated. Laboratory data were compared pre-to-post using paired-sample t-tests using a false discovery rate correction for the family of tests. Analyses were conducted in *R* with two-tailed tests and family-wise type I error rate of 0.05.

## Results

### Sample characteristics

Participants were recruited from June 2021 through March 2022. The trial was discontinued after the research and development arm of Canopy Growth (the sponsor company) was closed. A total of 11 male and 8 female participants completed the study. Sample characteristics are included in Table 1. The sample predominantly consisted of white, non-Hispanic, male individuals. Average number of drinks per week at baseline was 11.1 with a standard deviation of 11.3 drinks. Please refer to full CONSORT flow diagram.

**Table 1:** Sample characteristics (*n* = 19)

	Mean (SD)/%	Range
Age (Years)	34.2 (14.8)	21–64
Sex^a^		
Male	57.9% (*n* = 11)	
Female	42.1% (*n* = 9)	
Race		
White	73.7% (*n* = 14)	
Asian	5.3% (*n* = 1)	
African American	10.5% (*n* = 2)	
Other Ethnicity	10.5% (*n* = 2)	
Hispanic or Latino	15.8% (*n* = 3)	
Not Hispanic or Latino	84.2% (*n* = 16)	
Body Mass Index (BMI)	28.4 (SD = 7.1)	19.3–42.5
Usual Alcohol Drinks/Week	11.1 (SD = 11.3)	0–43
Max Number of Drinks/Occasion	8.2 (SD = 6.2)	2–24
Binge Drinking Occasions/Year	19.2 (SD = 36.8)	0–156

^a^All participants’ gender identity was congruent with their biological sex

Results from the adapted AUDIT questionnaire are included in Table 2. As reported in the screening visit, all participants who were included in the analysis reported drinking at least “sometimes” and there was a range of how many drinks were consumed on a given day. Most participants denied feeling that their alcohol use was out of their control or regularly associated with significant impacts on mood, cognition or functioning. No participants reported injuries because of their drinking or receiving concerns from others about their drinking.

**Table 2:** Adapted AUDIT questions and responses/% of total sample (*n* = 19). % F = % female responses from total sample

1) How often do you have a drink containing alcohol?^a^	Never (0%)	Almost Never (0%)	Sometimes (37%; 26% F)	Fairly Often (53%; 5% F)	Very Often (10%; 10% F)
2) How many drinks containing alcohol do you have on a typical day when you are drinking?	1 or 2 (21%; 16% F)	3 or 4 (53%; 16% F)	5 or 6 (10%; 0% F)	7, 8, or 9 (16%; 10% F)	10 or more (0%)
3) How often do you have six or more drinks on one occasion?	Never (16%; 16% F)	Less than monthly (26%; 16% F)	Monthly (42%; 0%F)	Weekly (16%; 10% F)	Daily or almost daily (0%)
4) How often during the last year have you found that you were not able to stop drinking once you had started?	Never (95%; 37% F)	Less than monthly (5%; 5% F)	Monthly (0%)	Weekly (0%)	Daily or almost daily (0%)
5) How often during the last year have you failed to do what was normally expected from you because of drinking?	Never (95%; 42% F)	Less than monthly (5%; 0% F)	Monthly (0%)	Weekly (0%)	Daily or almost daily (0%)
6) How often during the last year have you needed a first drink in the morning to get yourself going after a heavy drinking session?	Never (100%; 42% F)	Less than monthly (0%)	Monthly (0%)	Weekly (0%)	Daily or almost daily (0%)
7) How often during the last year have you had a feeling of guilt or remorse after drinking?	Never (74%; 32% F)	Less than monthly (16%; 0% F)	Monthly (10%; 10% F)	Weekly (0%)	Daily or almost daily (0%)
8) How often during the last year have you been unable to remember what happened the night before because you had been drinking?	Never (53%; 21%F)	Less than monthly (37%; 16% F)	Monthly (5%; 5% F)	Weekly (0%)	Daily or almost daily (5%; 0% F)
9) Have you or someone else been injured as a result of your drinking?	No (100%)	Yes, but not in the last year (0%)	Yes, during the last year (0%)		
10) Has a relative or friend or a doctor or another health worker been concerned about your drinking or suggested you cut down?	No (100%)	Yes, but not in the last year (0%)	Yes, during the last year (0%)		

^a^ In the original AUDIT, the answers to item 1 are “Never”, “Monthly or less”, “2–4 times a month”, “2–3 times a week”, and “4 or more times a week.” Item 1 from the adapted AUDIT has the following responses: “Never”, “Almost Never”, “Sometimes”, “Fairly Often”, and “Very Often”. Because of this modification of item 1, the scoring system for the AUDIT was not applied. Instead, we indicate the percent of responses to each item

#### CONSORT 2025 flow diagram

Flow diagram of the progress through the phases of a randomised trial of two groups (that is, enrolment, intervention allocation, follow-up, and data analysis).
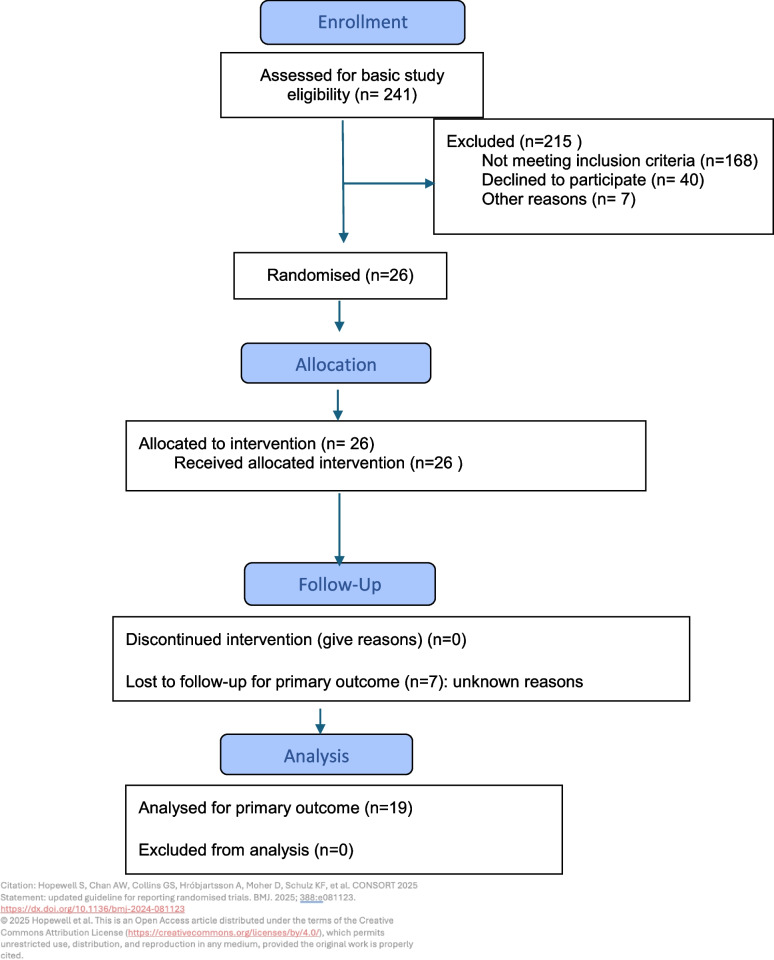


### Adverse events

CBD was well-tolerated in both the acute laboratory and chronic settings. No adverse events were recorded during any of the acute laboratory sessions. Two adverse events (somnolence and headache) were reported during the chronic portion of the study via the daily diary on Metricwire and resolved by next day report.

### Acute experimental sessions

**Fig. 2:**
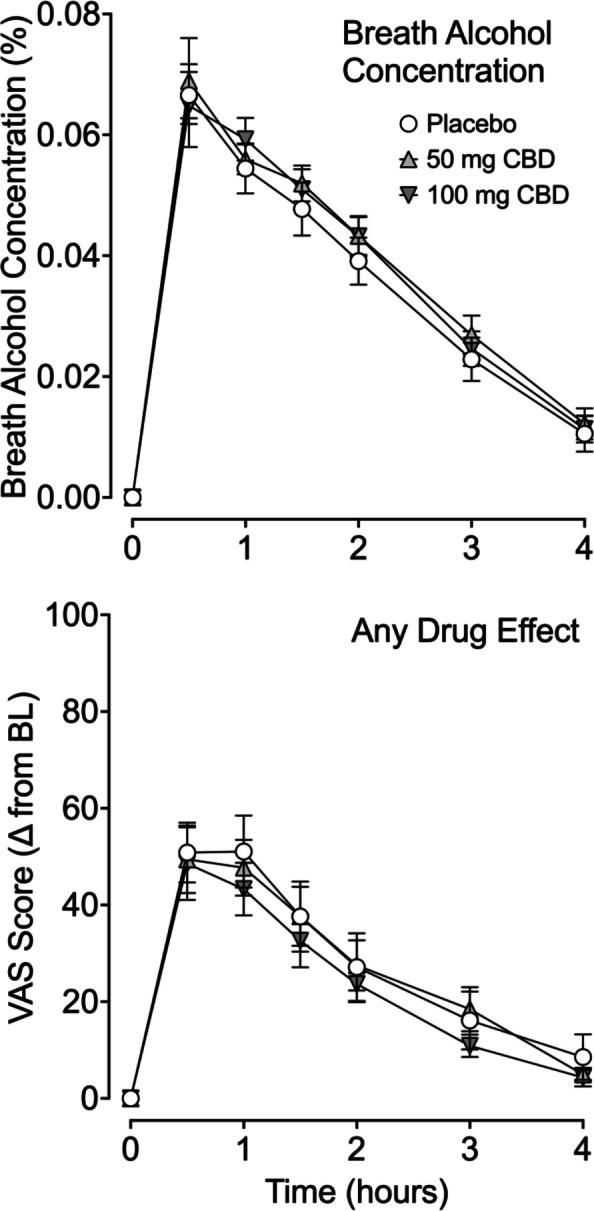
Effects of co-administration of CBD and alcohol on breath alcohol concentration and subjective drug effects during the laboratory session. Data are mean (SEM) breath alcohol concentrations (top panel) and change from baseline ratings of feeling any drug effect (bottom panel) for co-administration of alcohol plus placebo (circles), 50 mg CBD (triangles), and 100 mg CBD (upside down triangles). No significant differences were observed by CBD condition

**Fig. 3:**
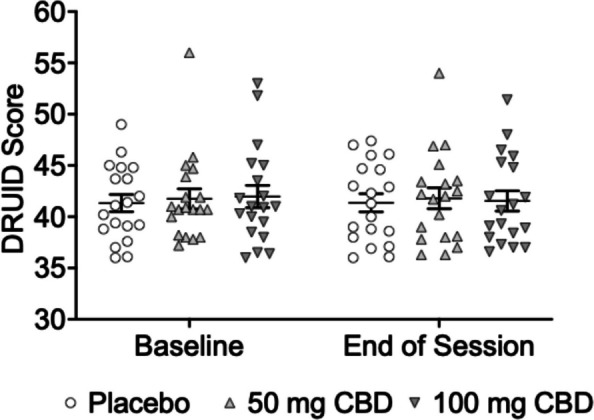
Performance effects of CBD and alcohol. Plotted are mean (SEM) and individual data points at baseline and end of session (240 min post administration) for DRUID performance after co-administration of placebo (circles), 50 mg CBD (triangles), and 100 mg CBD (upside down triangles). Higher scores indicate greater impairment. No significant differences were observed by CBD condition

For the acute experimental sessions, CBD and alcohol were administered to the participant by the study project investigator (PI) and research assistants and was adhered to in full by 19 participants. No significant differences in BrAC peak (*p* = 0.89 omnibus effect) or time course (interaction *p* = 0.82) were observed by CBD condition (Fig. 2). Table 3 contains average peak change from baseline for Alcohol Urge Questionnaire, Biphasic Alcohol Effects Scale, and Subjective Effects Questionnaire items. No significant effects of CBD dose were observed on peak change for these measures (*p* values > 0.05; see Fig. 2 for Any Effect and Supplemental Materials for model results). Similarly, no significant effects of CBD dose on timecourse or Tmax were observed (see Supplemental Table for Tmax descriptive statistics). DRUID scores were not significantly different between CBD conditions at baseline or end of session (4 h after alcohol administration, average BAL of 0.012; see Fig. 3)*.* No global DRUID score at the end of any session exceeded 57, a cutoff which has been used as a marker of impairment in prior studies (Spindle et al. 2021; Richman and May 2019).

**Table 3:** Laboratory drug challenge outcomes peak effects

	Placebo		50 mg CBD		100 mg CBD	
	Mean	SD	Mean	SD	Mean	SD
Breath Alcohol Concentration	0.071	0.014	0.074	0.026	0.072	0.025
Alcohol Urge Questionnaire	0.8	0.9	0.6	0.7	0.7	0.8
BAES Stimulatory	5.2	5.0	4.4	4.8	7.4	7.3
BAES Sedentary	13.1	11.9	14.3	12.1	15.0	9.2
Want Drink	1.4	1.7	0.7	0.9	1.4	1.9
Drug Effect Questionnaire						
Any Effect	58.8	28.3	54.9	25.9	53.0	28.4
Like	35.1	23.9	49.4	29.2	36.3	34.5
Dislike	31.8	34.0	21.8	28.0	26.8	32.1
Take Again	20.2	25.5	20.8	24.1	16.5	24.7
Anxious	4.8	9.8	8.3	11.9	11.8	20.1
Relaxed	10.9	16.5	16.9	18.7	20.1	22.9
Sleepy	34.5	24.6	45.7	28.1	37.6	23.5
Alert	8.3	15.9	9.6	13.9	9.9	13.1
Irritable	3.5	4.9	2.2	3.7	3.2	4.6
Restless	7.8	12.9	10.8	15.5	6.8	13.7
Happy	10.2	12.5	13.5	15.4	10.1	12.5
Sad	3.6	4.3	2.3	3.3	1.7	2.8

Values are peak effects. No significant differences were observed by CBD condition

### Chronic dosing

**Fig. 4:**
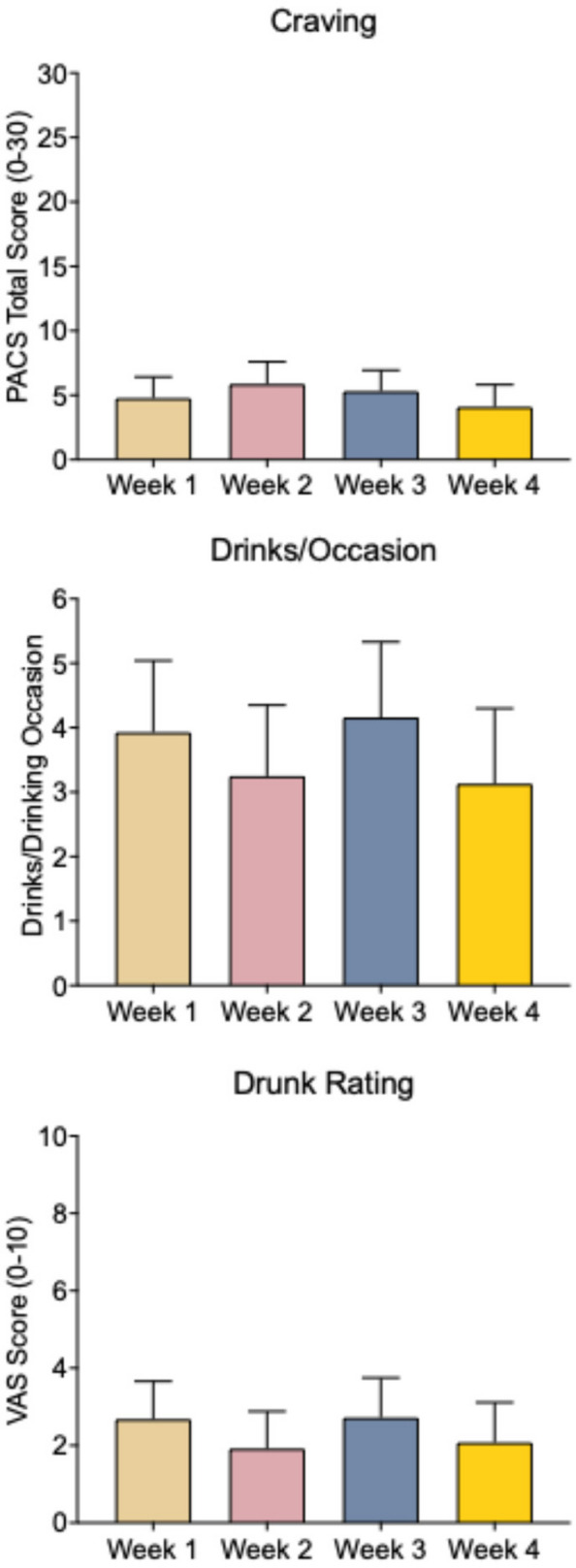
Craving, drinks/drinking day, and “drunk” ratings on drinking days over the 4-week outpatient period. Presented are estimated marginal means and confidence intervals estimated from linear mixed effect models for study variables over the 4-week outpatient period binned by study week

Adherence to reporting on Metricwire was high with an average of 85% completion rate for the 4-week period of data collection (range = 54%−100%). Self-reported adherence to study drug was also high, with participants completing an average of 92% of all assigned doses (range = 61%−100%). The self-report results are supported by detectable serum levels of CBD-COOH, a CBD metabolite with a particularly long half-life whose accumulation best indicates habitual use (Taylor et al. 2018), for each participant who presented for follow-up, with an average plasma level of 287.31 ng/mL (SD = 175.31). Alcohol use was reported in 45% of daily diary reports with an average of 3.8 (SD = 3.0) standard drinks consumed and “drunk” rating of 2.2 (SD = 2.6) on a scale of 0 to 10. Figure 4 contains estimated marginal means for alcohol craving, drinks/drinking day, and “drunk” ratings over the 4-week period by week of assessment. No significant differences from Week 1 were observed in alcohol craving, drinks/drinking day, or “drunk” ratings over the 4-week period (*p* values > 0.05 for main effect of Week in linear mixed effect models) indicating no significant change during the outpatient CBD administration period.

Table 4 laboratory chemistries at screening and the end-of-study follow up. A small magnitude decrease in protein (corrected *p* = 0.006) and globulin (corrected *p* = 0.014) were observed with no significant change in other laboratory values from screening.

**Table 4:** Laboratory chemistries at screening and end of study

	**Screening**		**End of Chronic Dosing**		
	Mean	SD	Mean	SD	*p*_fdr_
Weight (kg)	87.05	20.70	84.50	22.16	.521
BMI	28.40	7.13	27.36	6.70	.521
Glucose (mg/dL)	90.11	13.68	88.68	14.50	.755
Blood urea nitrogen – BUN (mg/dL)	12.95	5.36	12.95	3.39	.960
Creatinine (mg/dL)	0.92	0.19	0.89	0.13	.480
eGFR (mL/min/1.73)	101.74	18.61	102.84	15.12	.717
BUN/Creatine Ratio	14.11	5.05	14.79	3.97	.415
Sodium (mmol/L)	140.21	2.39	139.63	2.24	.486
Potassium (mmol/L)	4.41	0.48	4.32	0.35	.521
Chloride (mmol/L)	101.84	1.98	102.68	2.40	.120
CO2 (mmol/L)	23.68	3.04	22.63	2.69	.415
Calcium (mg/dL)	9.68	0.38	9.58	0.34	.644
Protein (g/dL)	**7.14**	**0.56**	**6.91**	**0.50**	.006**
Albumin (g/dL)	4.63	0.32	4.55	0.33	.273
Globulin (g/dL)	**2.51**	**0.32**	**2.36**	**0.31**	.014*
A/G Ratio	1.88	0.23	1.96	0.27	.182
Total Bilirubin (mg/dL)	0.34	0.18	0.47	0.28	.083
Alkaline Phosphatase—ALP (IU/L)	71.79	17.98	69.39	19.74	.521
Aspartate Aminotransferase—AST (IU/L)	23.63	6.72	21.26	4.59	.181
Alanine Aminotransferase—ALT (IU/L)	23.11	12.13	20.16	8.51	.309

^*^: *p* < 0.05. **: p < *0.01*

## Discussion

CBD use rates have increased markedly over the last few years, with one survey study identifying a 50% increase between 2019 and 2024 (Wilson-Poe et al. 2023). The rise in CBD use suggests a resultant rise in CBD and alcohol co-use. The co-use of CBD and alcohol is expected to increase as CBD product availability and use become more prevalent. Therefore, understanding the safety profile of CBD-alcohol co-use—including CBD use in the setting of regular outpatient alcohol consumption—represents an important public health goal. This Phase I study provides new data on the safety of CBD-alcohol co-use via examinations of co-administration of 50–100 mg CBD and alcohol (BAC of 0.06 g/100 mL) under controlled administration conditions in the laboratory and under conditions of chronic use of CBD over a 4-week period in healthy adults who engaged in moderate alcohol use. Participants tolerated use of CBD under both dosing conditions, with minimal adverse effects or changes in liver function. Additionally, CBD did not alter breath alcohol concentrations or the pharmacodynamic effects of alcohol, and did not affect craving or alcohol use patterns.

The lack of adverse effects from the acute laboratory sessions is consistent with the results from previous laboratory studies evaluating the subjective effects of alcohol and CBD co-administration (Belgrave et al. 1979; Karoly et al. 2023). The current project extends these findings to a four-week chronic CBD dosing period that likewise did not identify appreciable adverse effects outside of a headache and somnolence—findings that could have arisen from a number of etiologies outside of CBD use and resolved the following day. Metabolic lab results likewise did not show changes that could indicate adverse effects. The stable levels of the liver enzymes ALT and AST suggest an absence of hepatotoxicity; this is particularly notable given the concerns about hepatoxicity from CBD, as have been previously stated (Lo et al. 2023), and alcohol’s well-documented effects on liver function (Lieber and Decarli 1991). The current findings are in contrast to a recent study by Florian and colleagues (2025) that reported elevated liver enzyme levels greater than 3 times the upper limit of normal in 8 of the 201 healthy adults using a higher daily CBD dose (5 mg/kg or 350 mg total dose) for 4 weeks (Florian et al. 2025). The lack of effects of 50 or 100 mg CBD on LFT levels and liver integrity in the current study could be due to the lower dose tested or to the small number of participants included in the final analysis (*n* = 19) as the targeted number of participants determined in the original power analysis was 30. Future studies involving sample sizes at least as large as the one generated in the power analysis are needed to verify lack of 50–100 mg/day CBD on liver function among individuals with moderate alcohol use. Since the current study excluded those with severe AUD, it cannot be concluded from this project whether CBD offers any protection against alcohol-induced liver injury as has been seen in other studies (Yang et al. 2014; Wang et al. 2017). Future projects involving comparison of LFT changes between people with severe AUD randomized to either CBD or placebo could help address this question.

The only statistically significant changes in laboratory values over the 4-week outpatient period were very small decreases in total protein and globulins. It is unlikely that these findings are attributable to CBD or alcohol given that globulin levels follow a U-shaped curve in relation to alcohol use (Imhof et al. 2001), and CBD’s effects on total protein and globulin have received minimal attention in the literature. Should these changes be related to CBD use, their small magnitude was not clinically significant and unlikely to pose any major clinical concerns. These data therefore suggest that CBD, when used at the current doses (up to 100 mg daily), is likely to be well-tolerated and safe among healthy adults drinking moderate amounts of alcohol.

The present study also demonstrated no association between CBD administration and alcohol pharmacokinetics. The lack of a significant difference between conditions with respect to peak BrAC contrasts with results from Consroe et al. (1979) that illustrated statistically significant reductions in peak blood alcohol concentrations with CBD and alcohol vs alcohol alone. The CBD doses used in the study by Consroe and colleagues (1979) (200 mg) were higher than those in this study (50 mg and 100 mg), suggesting that higher doses may be needed to observe changes in peak BrAC. In another study, Karoly et al. (2023) demonstrated that administration of CBD doses as low as 30 mg were associated with a slower decline in BrAC compared to placebo, which contrasts with this study’s lack of observed effects of CBD on BrAC over time. As the effects of CBD on the time course of alcohol concentration from the study by Karoly and colleagues (2023) were small and lacked precision; study differences with respect to CBD’s impact on alcohol’s pharmacokinetics may stem from variation in statistical analysis. Future studies with larger sample sizes and varied doses of CBD are needed to better understand the effect of CBD on alcohol’s pharmacokinetics.

Other measures administered during the acute laboratory session, including the BAES, the AUQ, and the DRUID, likewise did not demonstrate changes associated with CBD administration at either CBD dose compared to placebo. The absence of an observed effect on alcohol craving in our study mirrors outcomes from Karoly et al. (2023) that likewise did not observe an association of coadministration of CBD and alcohol with changes in AUQ scores. Yet the lack of statistically significant findings in our study is in contrast to results from Karoly et al. (2023) that did identify a statistically significant delay in the decline of the Sedentary component of the BAES. However, Karoly et al. acknowledged that these differences were too small to suggest that CBD would have a meaningful effect on subjective effects mediated by alcohol; this interpretation is largely in agreement with our own conclusion that CBD does not substantially impact subjective effects that arise from alcohol at the doses studied. Higher doses of CBD or different formulations (e.g., inhaled or sublingual) may yield different alcohol-related effects.

Regarding alcohol-related impairment, Consroe et al. (1979) found that CBD-alcohol co-administration significantly impacted cognition and motor skills compared to placebo, which differs from the our study that did not identify any effect on impairment. The measures used in the Consroe study, including the differential aptitude test (Anastasi 1969), cancellation test (Sack and Rice 1974), time production test (Karniol et al. 1974), and finger tap test (Halstead 1947), differed from the measure used in our study (the DRUID). Notably, our study did not examine the timecourse of any effects of CBD and alcohol on the DRUID; future studies should assess objective global impairment at multiple timepoints after dosing of CBD and alcohol to understand the extent and timing of any potential impairment associated with co-use of CBD and alcohol.

Our study did not observe significant effects of CBD on alcohol use patterns in the outpatient setting. The lack of an effect of CBD on alcohol use is a novel finding as this is the first study to explore co-use of CBD and alcohol in a real-world setting. An earlier study by Karoly et al. (2021) found that CBD-dominant cannabis was associated with fewer drinking days and fewer drinks on drinking days compared to either THC-dominant or balanced THC-CBD strains. It is difficult to interpret these results against those of our study because the chronic dosing portion of our study did not involve comparisons between groups taking different forms of cannabis. A future study assessing the effects of CBD isolate relative to whole-plant CBD-dominant strains on alcohol use behavior could be important for clarifying any influence that minor phytocannabinoids or terpenes may have on alcohol use patterns.

It bears reiterating that our study did not include individuals with diagnosed AUD but rather focused on healthy individuals with moderate alcohol use. As such, our population would have low baseline craving and less room for intervention (i.e., floor effect). CBD’s anti-craving potential may be more apparent, then, among individuals with AUD, as was shown in a recent study completed by Zimmerman et al. (2025). In the Zimmerman et al. study, 800 mg CBD administered to individuals with mild-to-severe AUD was associated with significantly reduced cravings induced by acute stress and alcohol-cues in the laboratory. Additionally, the relatively low dose of CBD exposure in our study may have limited its potential to modulate alcohol use patterns. Future studies with higher CBD doses and in populations with higher baseline craving levels may help clarify the extent to which CBD influences alcohol use patterns.

Findings from this work need to be interpreted in the setting of certain limitations. As mentioned earlier, our final sample size was less than the sample size calculate by the power analysis, and thus there is reason to believe that the sample may be underpowered to identify significant differences. The sample demographics, consisting of over 80% white individuals and nearly 60% male individuals, are not representative of the US population (Roman 2022), impacting external validity. Although participants were asked about medications or supplements initiated during the chronic phase of the study, use of illicit substances (i.e. nonprescribed opioids, cocaine, etc.) was not assessed. The use of such substances would be unlikely in this population given use of such substances prior to the study represented one of the exclusion criteria, but even occasional use that remains unmeasured could affect subjective outcomes. Lastly, the lack of a placebo condition in the outpatient phase of the study prevents any between-subject comparisons of CBD on pharmacodynamic outcomes related to CBD and alcohol co-use. The strengths of this study balance its limitations. The finding that CBD and alcohol co-administration did not produce significant adverse effects or changes in subjective effects aligns with previous research, which indicates that the combination is well-tolerated among otherwise healthy individuals with moderate alcohol use. Another notable aspect of this study is the use of the chronic dosing outpatient phase, offering valuable insights into the real-world safety of CBD and alcohol co-use.

## Conclusions

Ultimately, this Phase I study provides evidence that CBD administered at or below the 100 mg daily dose that has been identified as a maximum safe daily dose for healthy, non-pregnant or lactating adults, is likewise safe and tolerable among healthy adults who drink moderate amounts of alcohol (Henderson et al. 2023b). In addition, CBD also did not adversely affect their alcohol use patterns or craving for alcohol. By exploring the safety profile of concurrent use of alcohol and CBD at doses commonly taken in the general population, these results provide salient health information on an increasingly prevalent behavior. These findings should be verified in future studies with larger sample populations. Future studies could also explore higher CBD doses among patients with heavy alcohol use or diagnosed AUD to determine its safety and impacts on cravings and alcohol use patterns in these populations.

## Supplementary Information

Additional file 1. CONSORT Checklist (completed CONSORT checklist).
Additional file 2. Subjective Effects Questionnaire (copy of questionnaire given to participants to assess subjective responses to study drugs during laboratory sessions).

Additional file 3. Supplemental tables indicating the Mixed Effects Model Results from Acute and Chronic Dosing Sessions

Additional file 4. Supplemental Table indication Time to Peak Effects (Tmax) Across Laboratory Drug Challenge Outcomes

## Data Availability

The data that support the findings of this study are available from the corresponding author upon reasonable request.

## Abbreviations

ALT: Alanine aminotransferase
AST: Aspartate aminotransferase
AUD: Alcohol Use Disorder
AUQ: Alcohol Urge Questionnaire
BAC: Blood alcohol concentration
BAES: Biphasic Alcohol Effects Scale
BrAC: Breath alcohol content
CBD: Cannabidiol
CMP: Comprehensive metabolic panel
DRUID: DRiving Under the Influence of Drugs®
MCT: Medium-chain triglyceride
PACS: Penn Alcohol Craving Scale
THC: Delta-9-tetrahydrocannabinol
TLFB: Timeline Followback
