# Supplementary material for: Safety of Co-Administered Cannabidiol (CBD) and alcohol: a Phase I study
Source: J Cannabis Res. 2026 Jun 13;8:84. doi: 10.1186/s42238-026-00457-1 (PMC13352867; doi:10.1186/s42238-026-00457-1)
Supplement: Supplementary file 1 — Additional file 1. CONSORT Checklist (completed CONSORT checklist). Additional file 2. Subjective Effects Questionnaire (copy of questionnaire given to participants to assess subjective responses to study drugs during laboratory sessions). [file 42238_2026_457_MOESM1_ESM.docx]

**Supplemental Figure 1: Subjective Effects Questionnaire**

INSTRUCTIONS: This questionnaire asks you how you are feeling after taking the study product and beverage that were given to you. Please mark on the line to show how strongly you are feeling each of the following effects **right now**.

[*This rating scale is a visual analog scale ranging from 0 (not at all) to 100 (Extremely) that is usually completed on a computer. Using a mouse, the participant places the cursor on the line and clicks on a sliding scale to indicate how they are feeling “right now”.]*

1. Do you feel an effect from the study product and beverage right now?

| Not at all |  | Extremely |
| --- | --- | --- |
|  |  |  |
|  |  |  |

1. Do you like any of the effects that you are feeling right now?

| Not at all |  | Extremely |
| --- | --- | --- |
|  |  |  |

1. Do you dislike any of the effects that you are feeling right now?

| Not at all |  | Extremely |
| --- | --- | --- |
|  |  |  |

1. How likely would you be to take this study product and beverage again?

| Not at all |  | Extremely |
| --- | --- | --- |
|  |  |  |

1. Do you feel anxious or nervous right now?

| Not at all |  | Extremely |
| --- | --- | --- |
|  |  |  |

1. Do you feel relaxed right now?

| Not at all |  | Extremely |
| --- | --- | --- |
|  |  |  |
|  |  |  |

1. Do you feel sleepy or tired right now?

| Not at all |  | Extremely |
| --- | --- | --- |
|  |  |  |
|  |  |  |
|  |  |  |

1. Do you feel alert right now?

| Not at all |  | Extremely |
| --- | --- | --- |
|  |  |  |
|  |  |  |

1. Do you feel irritable right now?

| Not at all |  | Extremely |
| --- | --- | --- |
|  |  |  |

1. Do you feel restless right now?

| Not at all |  | Extremely |
| --- | --- | --- |
|  |  |  |

1. Do you feel happy right now?

| Not at all |  | Extremely |
| --- | --- | --- |
|  |  |  |
|  |  |  |

1. Do you feel sad right now?

| Not at all |  | Extremely |
| --- | --- | --- |
