# Supplementary material for: Safety of Co-Administered Cannabidiol (CBD) and alcohol: a Phase I study
Source: J Cannabis Res. 2026 Jun 13;8:84. doi: 10.1186/s42238-026-00457-1 (PMC13352867; doi:10.1186/s42238-026-00457-1)
Supplement: Supplementary file 2 — Additional file 3. Supplemental tables indicating the Mixed Effects Model Results from Acute and Chronic Dosing Sessions [file 42238_2026_457_MOESM2_ESM.docx]

Supplemental Table 1. Mixed Effect Model Results from Acute Sessions

|  | 50 mg CBD | | |  | 100 mg CBD | | |
| --- | --- | --- | --- | --- | --- | --- | --- |
| Peak Effects | b | t | p |  | b | t | p |
| BAC | 0.003 | 0.46 | 0.65 |  | 0.001 | 0.12 | 0.90 |
| AUQ | -0.20 | -1.15 | 0.26 |  | -0.07 | -0.41 | 0.69 |
| BAES Stimulatory | -0.74 | -0.44 | 0.66 |  | 2.26 | 1.37 | 0.18 |
| BAES Sedentary | 1.21 | 0.59 | 0.56 |  | 1.89 | 0.92 | 0.37 |
| Want Drink | -0.74 | -1.80 | 0.08 |  | -0.05 | -0.13 | 0.90 |
| ***DEQ*** | | |  |  |  |  |  |
| Any Effect | -3.84 | -0.73 | 0.47 |  | -5.79 | -1.1 | 0.28 |
| Like | 14.32 | 1.83 | 0.08 |  | 1.21 | 0.15 | 0.88 |
| Dislike | -9.95 | -1.53 | 0.14 |  | -4.95 | -0.76 | 0.45 |
| Take Again | 0.58 | 0.10 | 0.92 |  | -3.68 | -0.61 | 0.54 |
| Anxious | 3.47 | 0.86 | 0.40 |  | 7.05 | 1.75 | 0.09 |
| Relaxed | 6.00 | 1.07 | 0.29 |  | 9.16 | 1.63 | 0.11 |
| Sleepy | 11.21 | 1.59 | 0.12 |  | 3.11 | 0.44 | 0.66 |
| Alert | 1.32 | 0.32 | 0.75 |  | 1.63 | 0.39 | 0.70 |
| Irritable | -1.32 | -1.02 | 0.31 |  | -0.26 | -0.20 | 0.84 |
| Restless | 3.05 | 0.94 | 0.35 |  | -0.95 | -0.29 | 0.77 |
| Happy | 3.32 | 0.91 | 0.37 |  | -0.16 | -0.04 | 0.97 |
| Sad | -1.32 | -1.37 | 0.18 |  | -1.89 | -1.97 | 0.06 |

Note. BAC = Breath Alcohol Concentration; AUC = Alcohol Urge Questionnaire; BAES = Biphasic Alcohol Effects Scale; DEQ = Drug Effects Questionnaire. Results presented are parameter estimates for peak effects after CBD dosing relative to the reference group of 0 mg CBD.

Supplemental Table 2. Mixed Effect Model Results for Chronic Dosing Models

|  | Week 2 | | |  | Week 3 | | |  | Week 4 | | |
| --- | --- | --- | --- | --- | --- | --- | --- | --- | --- | --- | --- |
|  | b | t | p |  | b | t | p |  | b | t | p |
| “Drunk” Rating | -0.76 | -1.86 | 0.06 |  | 0.04 | 0.08 | 0.93 |  | -0.6 | -1.37 | 0.17 |
| Drinks/Drinking Day | -0.68 | -1.5 | 0.14 |  | 0.23 | 0.48 | 0.63 |  | -0.8 | -1.65 | 0.1 |
| Alcohol Craving | 1.1 | 1.23 | 0.23 |  | 0.54 | 0.64 | 0.52 |  | -0.68 | -0.76 | 0.45 |

Note. Results presented are parameter estimates for Weeks 2-4 relative to the reference group of Week 1.
