# Supplementary material for: Safety of Co-Administered Cannabidiol (CBD) and alcohol: a Phase I study
Source: J Cannabis Res. 2026 Jun 13;8:84. doi: 10.1186/s42238-026-00457-1 (PMC13352867; doi:10.1186/s42238-026-00457-1)
Supplement: Supplementary file 3 — Additional file 4. Supplemental Table indication Time to Peak Effects (Tmax) Across Laboratory Drug Challenge Outcomes [file 42238_2026_457_MOESM3_ESM.docx]

Supplemental Table 3: Laboratory Drug Challenge Outcomes Time to Peak Effects (Tmax)

|  | Placebo | | 50 mg CBD | | 100 mg CBD | |
| --- | --- | --- | --- | --- | --- | --- |
|  | Mean | SD | Mean | SD | Mean | SD |
| Breath Alcohol Concentration | 36.3 | 12.6 | 47.4 | 27.0 | 47.4 | 20.8 |
| Alcohol Urge Questionnaire | 64.7 | 55.9 | 64.7 | 52.2 | 60.0 | 52.0 |
| BAES Stimulatory | 82.1 | 86.5 | 53.7 | 52.5 | 77.4 | 68.1 |
| BAES Sedentary | 82.1 | 52.8 | 91.6 | 62.8 | 97.9 | 68.4 |
| Want Drink |  |  |  |  |  |  |
| **Drug Effect Questionnaire** | 55.3 | 52.2 | 50.5 | 48.0 | 53.7 | 57.9 |
| Any Effect | 60.0 | 51.4 | 58.3 | 37.8 | 45.0 | 21.2 |
| Like | 68.3 | 53.3 | 98.3 | 62.4 | 91.7 | 71.6 |
| Dislike | 120.0 | 82.3 | 98.8 | 62.5 | 77.6 | 60.1 |
| Take Again | 55.3 | 51.2 | 80.5 | 73.5 | 60.0 | 49.0 |
| Anxious | 88.3 | 73.8 | 80.0 | 69.0 | 83.3 | 64.7 |
| Relaxed | 112.1 | 83.6 | 77.4 | 50.3 | 88.4 | 63.6 |
| Sleepy | 134.2 | 68.1 | 118.4 | 82.1 | 118.4 | 61.2 |
| Alert | 123.2 | 78.0 | 115.3 | 80.2 | 135.8 | 92.4 |
| Irritable | 70.0 | 43.7 | 61.7 | 46.6 | 96.7 | 73.2 |
| Restless | 101.7 | 81.8 | 85.0 | 76.5 | 81.7 | 76.2 |
| Happy | 78.9 | 65.7 | 88.4 | 82.7 | 85.3 | 73.7 |
| Sad | 88.3 | 67.1 | 95.0 | 76.5 | 93.5 | 80.7 |

Note. Values are time to peak effects. Units are in minutes. No significant differences were observed by CBD condition.
